# Lignin Induces ES Cells to Differentiate into Neuroectodermal Cells through Mediation of the Wnt Signaling Pathway

**DOI:** 10.1371/journal.pone.0066376

**Published:** 2013-06-21

**Authors:** Yu Inoue, Seiji Hasegawa, Takaaki Yamada, Yasushi Date, Hiroshi Mizutani, Satoru Nakata, Hirohiko Akamatsu

**Affiliations:** 1 Research Laboratories, Nippon Menard Cosmetic Co., Ltd., Aichi, Japan; 2 Department of Dermatology, Fujita Health University School of Medicine, Aichi, Japan; 3 Department of Applied Cell and Regenerative Medicine, Fujita Health University School of Medicine, Aichi, Japan; National Cancer Institute, United States of America

## Abstract

Embryonic stem cells (ES cells) are characterized by their pluripotency and infinite proliferation potential. Ever since ES cells were first established in 1981, there have been a growing number of studies aimed at clinical applications of ES cells. In recent years, various types of differentiation inducement systems using ES cells have been established. Further studies have been conducted to utilize differentiation inducement systems in the field of regenerative medicine. For cellular treatments using stem cells including ES cells, differentiation induction should be performed in a sufficient manner to obtain the intended cell lineages. Lignin is a high-molecular amorphous material that forms plants together with cellulose and hemicelluloses, in which phenylpropane fundamental units are complexly condensed. Lignin derivatives have been shown to have several bioactive functions. In spite of these findings, few studies have focused on the effects of lignin on stem cells. Our study aimed to develop a novel technology using lignin to effectively induce ES cells to differentiate into neuroectodermal cells including ocular cells and neural cells. Since lignin can be produced at a relatively low cost in large volumes, its utilization is expected for more convenient differentiation induction technologies and in the field of regenerative medicine in the future.

## Introduction

Embryonic stem cells (ES cells) are characterized by their pluripotency and infinite proliferation potential [1]. Ever since ES cells were first established in 1981, there have been a growing number of studies focusing on clinical applications of ES cells. In recent years, various types of differentiation inducement systems using ES cells have been established [2], [3]. Further studies have been conducted to utilize differentiation inducement systems in the field of regenerative medicine [4].

For cellular treatments using stem cells including ES cells, differentiation induction should be performed in a sufficient manner to obtain the intended cell lineages. Various kinds of compounds have been proven to control the differentiation of ES cells. For example, retinoic acid (RA) is known to notably promote the differentiation of ES cells into neural cells [5]. Takahashi T et al. demonstrated that ascorbic acid promotes the differentiation of ES cells into cardiomyocytes [6]. Nagafune et al. also found that (-)-indolactam V promotes the differentiation of ES cells into pancreas progenitor cells [7]. Furthermore, some botanical ingredients have been reported to have the potential to control the differentiation of stem cells including red ginseng extract, which has been demonstrated to promote the differentiation of ES cells into mesendoderm cell lineage cells [8]. Reynertson et al. performed screenings by adding a variety of medicinal plants extracts to ES cells and discovered various extracts that control the differentiation of ES cells [9]. In this study, we focused on lignin, which exists abundantly in nature.

Lignin is a high-molecular amorphous material that forms plants together with cellulose and hemicelluloses, in which phenylpropane fundamental units are complexly condensed. Lignin derivatives have been shown to have several bioactive functions. Ito Y et al. reported that lignin derivatives suppress the apoptosis of neural cells caused by oxidative stress such as active oxygen [10]. In spite of these findings, few studies have focused on the effects of lignin on stem cells.

In our study, we added lignin to ES cells and evaluated its effects on the differentiation of ES cells. Results showed that lignin decreased the expression of undifferentiation markers and promoted the expression of neuroectodermal markers, while simultaneously markedly downregulating the expression of Wnt target genes. Furthermore, it was demonstrated that when lignin was added to a melanocyte differentiation inducement system, differentiation into ocular cells was promoted. Since these effects were recovered when 6-BIO, a Wnt/β catenin signaling pathway activator, was added, it was suggested that lignin induces ES cells to differentiate into neuroectodermal cells through mediation of the Wnt/β catenin signaling pathway.

Our study aimed to develop a novel technology using lignin to effectively induce ES cells to differentiate into neuroectodermal cells including ocular cells and neural cells. Since lignin can be produced at a relatively low cost in large volumes, its utilization is expected for more convenient differentiation induction technologies and in the field of regenerative medicine in the future.

## Materials and Methods

### Cell Culture

BRUCE-4 ES cells (MILLIPORE, Billerica, MA), derived from mouse ES cells of the cell line C57/BL6J, were maintained in Dulbecco’s modified Eagle medium (Invitrogen Corp., Carlsbad, CA) supplemented with 15% Fetal Bovine Serum (FBS) (Sigma, St. Louis, MO), ES Cell Qualified L-Glutamine Solution (CHEMICON International, Inc., Temecula, CA), ES Cell Qualified 2-Mercaptoethanol (CHEMICON), ES Cell Qualified Non Essential Amino Acids (CHEMICON), ES Cell Qualified Nucleosides (CHEMICON), and Leukemia inhibitory factor (LIF) (CHEMICON), according to the manufacturer’s protocol. Mouse embryonic fibroblasts (MEF) (MILLIPORE) treated with 10 µg/mL of Mitomycin C (Sigma) were used as feeder layer cells.

For induction of differentiation into melanocytes, ST2 cells (Riken Cell Bank, Ibaraki, Japan) were used as feeder layer cells. ES cells were seeded on ST2 feeder layer cells in 24-well plates (500 cells/well), and were cultured in Minimum Essential Medium Alpha Modification (Invitrogen) supplemented with 10% FBS, 100 nM dexamethasone (Sigma), 20 pM basic fibroblast growth factor (PeproTech Inc., Rocky Hill, NJ, USA), 10 pM cholera toxin (Bio Academia, Osaka, Japan), and 100 ng/mL Endothelin 3 (Wako Pure Chemical Industries, Co. Ltd., Osaka, Japan).

For induction of differentiation into neuronal cells, ST2 cells were also used as feeder layer cells. ES cells were seeded on ST2 feeder layer cells in 24-well plates (500 cells/well), and were cultured in DMEM supplemented with 10% KnockOut. Serum Replacement (Invitrogen), 2 mM L-Glutamine, 1 mM sodium pyruvate (sigma), 0.1 mM 2-Mercaptoethanol (sigma), 0.1 mM Non Essential Amino Acids (Invitrogen). Lignin (Alkaline) (Tokyo Chemical Industry Co., Ltd. Tokyo, Japan) was dissolved in water at a concentration of 5 mg/mL as a stock solution, and was then added to cell cultures at a final concentration of 12.5–50 µg/mL.

### Immunocytochemistry

ES cells were washed with phosphate-buffered saline (PBS), fixed in 4% paraformaldehyde in PBS for 1 hr at 4°C, and permeabilized in PBS containing 0.25% Triton X-100 (Sigma) and 1% bovine serum albumin (Sigma) for 30 min before the detection of ocular and neuronal cell markers with immunofluorescence. The media with anti alpha-B crystallin rabbit polyclonal antibody (Santa Cruz Biotechnology,Inc., Santa Cruz, CA), anti Rpe65 mouse monoclonal antibody(Novus Biologicals, Inc., Littleton, CO), anti Keratin anti rabbit polyclonal antibody (LSL Corp., Tokyo, Japan), anti ZO-1 goat polyclonal antibody (Santa Cruz Biotechnology) anti Neurofilament rabbit polyclonal antibody (CHEMICON) and anti MAP2 rabbit polyclonal antibody (Sigma) After being washed in PBS, the cells were stained with Alexa Fluor 488 donkey anti-rabbit IgG (Molecular Probes Inc., Eugene, OR, USA), Alexa Fluor 594 donkey anti-mouse IgG (Molecular Probes), Alexa Fluor 568 goat anti-rabbit IgG (Molecular Probes), Alexa Fluor 594 donkey anti-goat IgG (Molecular Probes)Subsequently, cells were washed in PBS, and 1 µg/mL DAPI solution (DOJINDO Laboratories, Kumamoto, Japan) was added to confirm the presence of cell nuclei.

### Quantitative Real-time PCR Analysis

Total RNA was extracted from cells at various stages using TRIZOL® Reagent (Invitrogen), and cDNA was synthesized by reverse transcription. Real-time PCR was performed with the SuperScriptTM III Platinum® Two-Step qRT-PCR kit (Invitrogen), using the 7300 Real Time PCR System (Applied Biosystems, Tokyo, Japan) according to the manufacturer’s protocol. The primer sequences used were as follows:

Gapdh sense primer 5′-TGCACCACCAACTGCTTAGC-3′.

Gapdh anti-sense primer 5′- TCTTCTGGGTGGCAGTGATG-3′.

Nanog sense primer 5′-ATGCCTGCAGTTTTTCATCC-3′.

Nanog anti-sense primer 5′-GAGGCAGGTCTTCAGAGGAA-3′.

Rex1 sense primer 5′ - TGTGCTGCCTCCAAGTGTTG-3′.

Rex1 anti-sense primer 5′- ACTGATCCGCAAACACCTGC-3′.

Sox1 sense primer 5′- GCCGAGTGGAAGGTCATGT -3′.

Sox1 anti-sense primer 5′- TGTAATCCGGGTGTTCCTTCAT -3′.

Otx2 sense primer 5′- GAAAATCAACTTGCCAGAATCCA -3′.

Otx2 anti-sense primer 5′- GCGGCACTTAGCTCTTCGAT -3′.

Foxa2 sense primer 5′- GGCCCAGTCACGAACAAAGC -3′.

Foxa2 anti-sense primer 5′- CCCAAAGTCTCCACTCAGCCTC -3′.

Sox17 sense primer 5′- CAGAACCCAGATCTGCACAA -3′.

Sox17 anti-sense primer 5′- GCTTCTCTGCCAAGGTCAAC -3′.

Flk1 sense primer 5′- CTGTGGCGTTTCCTACTCCT -3′.

Flk1 anti-sense primer 5′- AGGAGCAAGCTGCATCATTT -3′.

Brachyury sense primer 5′- CAGCCCACCTACTGGCTCTA -3′.

Brachyury anti-sense primer 5′- GAGCCTCGAAAGAACTGAGC -3′.

Axin2 sense primer 5′- AGGATGTCTGGCAGTGGATGT -3′.

Axin2 anti-sense primer 5′- TTCTTATGCTTTGGGCACTATGG -3′.

Myc sense primer 5′- ATCAGCAACAACCGCAAGTG -3′.

Myc anti-sense primer 5′- GTGTCCGCCTCTTGTCGTTT -3′.

Lef1 sense primer 5′- AGGGCGACTTAGCCGACAT -3′.

Lef1 anti-sense primer 5′- GCTGGCTGGGATGATTTCG -3′.

Stat3 sense primer 5′- CCTGGCACCTTGGATTGAGA -3′.

Stat3 anti-sense primer 5′- CCAACGTGGCATGTGACTCT -3′.

Snail2 sense primer 5′- CTCCACTCCACTCTCCTTTACC -3′.

Snail2 anti-sense primer 5′- CTTGGATGAAGTGTCAGAGGAA -3′.

Twist sense primer 5′- AATTAGAAGAGCAGAGACCAAA -3′.

Twist anti-sense primer 5′- TGCCCCTCTGGGAATCT-3′.

Notch1 sense primer 5′- AACGTGGTCTTCAAGCGTGAT -3′.

Notch1 anti-sense primer 5′- AGCTCTTCCTCGTGGCCATA -3′.

P75 sense primer 5′- ATCTTGGCTGCTGTGGTTGTG -3′.

P75 anti-sense primer 5′- TATTTTGCTTGCAGCTGTTCCA -3′.

Six3 sense primer 5′- CCCTAGATCTCTATTCCTCCCACTTC -3′.

Six3 anti-sense primer 5′- GAAGTAGGGAGCAGTGGTGAGAA -3′.

Pax6 sense primer 5′- GCACCAAAGGGTCATCGC -3′.

Pax6 anti-sense primer 5′- TGGGGGGTGGATGGAAG -3′.

Cryg sense primer 5′- TGGCTGCTGGATGCTCTATG -3′.

Cryg anti-sense primer 5′- CCGCGACGCAGGAAGTA -3′.

Wnt1 sense primer 5′- AGCGTTCATCTTCGCAATCA -3′.

Wnt1 anti-sense primer 5′- AAATCGATGTTGTCACTGCAGC -3′.

Wnt3a sense primer 5′- CACCACCGTCAGCAACAGCC -3′.

Wnt3a anti-sense primer 5′- AGGAGCGTGTCACTGCGAAAG -3′.

Wnt8a sense primer 5′- ACGGTGGAATTGTCCTGAGCATG -3′.

Wnt8a anti-sense primer 5′- GATGGCAGCAGAGCGGATGG -3′.

Tuj1 sense primer 5′- CTCAAAATGTCATCCACCTT -3′.

Tuj1 anti-sense primer 5′- GTGAACTCCATCTCATCCAT -3′.

MAP2 sense primer 5′- ACTCAGCAACGTCTCATCTT -3′.

MAP2 anti-sense primer 5′- GTATTCACAAGCCCTGCTTA -3′.

The contents of the selected genes were normalized to Gapdh. All PCR products were checked by melting curve analysis to exclude the possibility of multiple products or incorrect product size. PCR analyses were conducted in triplicate for each sample.

### Statistical Analysis

Student’s T test was used for statistical analysis.

## Results

### 1. Effects of Lignin on the Expression of Differentiation and Undifferentiation Markers in Mouse ES Cells

**Figure 1:**
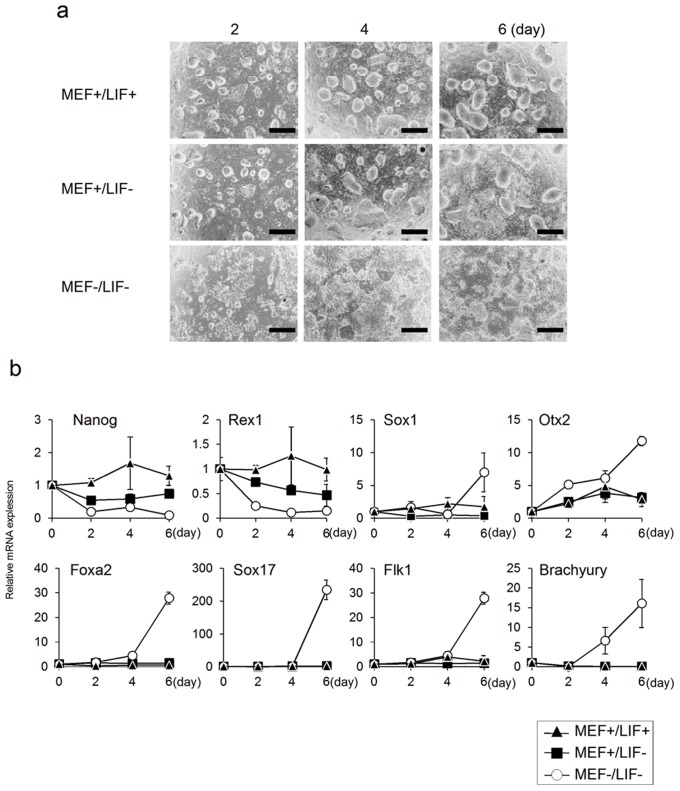
Time-course analysis of ES cells cultured under various conditions. (a) Microscopic images of ES cells. ES cells were cultured under various conditions (MEF+/LIF+, MEF+/LIF-, MEF−/LIF-). (b) Gene expression analysis by real-time PCR. Changes in the marker gene expressions associated with differentiation of ES cells were analyzed. The expression level of each marker gene were normalized to that on day 0. Scale bar: a = 200 µm. Data are expressed as the mean ± SD of the three experiments.

First, we cultured undifferentiated-state ES cells under three specific conditions (MEF+/LIF+, MEF+/LIF−, and MEF−/LIF−) and collected total RNAs on days 2, 4, and 6 to analyze the expression of differentiation and undifferentiation markers by real-time PCR (Fig. 1). When ES cells were cultured in the presence of MEF and LIF (MEF+/LIF+), they formed colonies, maintaining the expression of the undifferentiation markers, Nanog and Rex1. There were hardly any changes in the expression of the differentiation markers Sox1, Otx2, Foxa2, Sox17, Flk1, and Brachyury of each germ layer (Figs. 1a and b). When ES cells were cultured in the presence of MEF and absence of LIF (MEF+/LIF−), they formed colonies and showed little change in the expression of differentiation markers; however, there was decreased expression of the undifferentiation markers, Nanog and Rex1 (Figs. 1a and b). On the other hand, when ES cells were cultured in the absence of MEF and LIF (MEF−/LIF−), the expression of Nanog and Rex1 was notably decreased, and the expression of differentiation markers was significantly increased, without any ES cell colonies (Figs. 1a and b). These results indicated that ES cells remained undifferentiated in the presence of MEF and LIF, and that the expression of undifferentiation markers was decreased without increased expression of other differentiation markers in the presence of MEF and absence of LIF; therefore, ES cells remained relatively undifferentiated. It was also shown that when ES cells were cultured in the absence of MEF and LIF, the expression of undifferentiation markers was notably decreased, resulting in differentiation into triploblastic cells.

**Figure 2:**
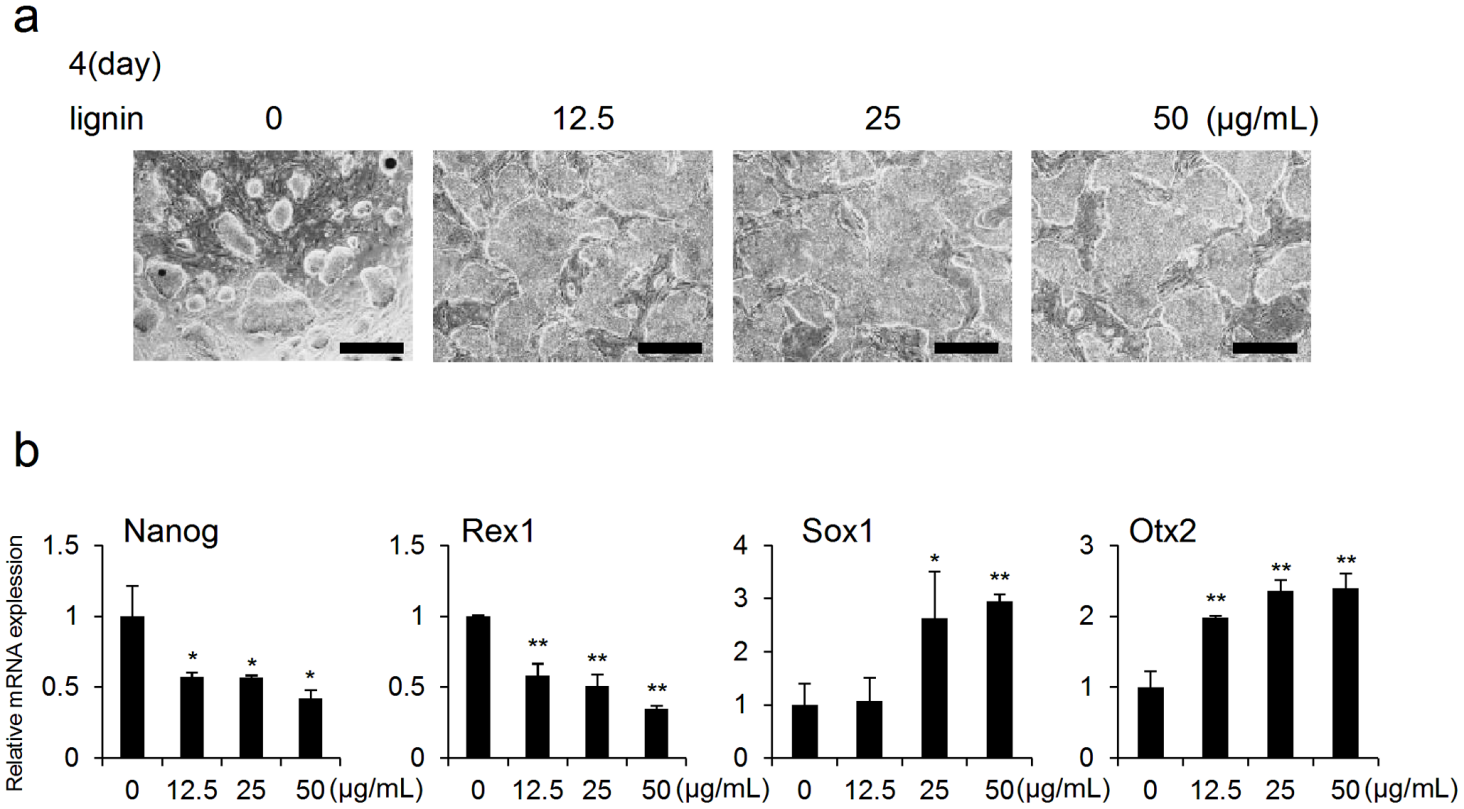
Analysis of the effects of lignin on ES cells cultured in presence of MEF and absence of LIF (MEF+/LIF−). (a) Microscopic images of ES cells on day 4 of culture. ES cells were cultured with lignin (12.5, 25 and 50 µg/mL). (b) Gene expression analysis by real-time PCR. Changes in the expressions of differentiation marker genes associated with the addition of lignin were analyzed on day 4 of culture. The expression level of each marker gene was normalized to control (0 µg/mL). Scale bar: a = 200 µm. Data are expressed as the mean ± SD of the three experiments. *P<0.05, **P<0.01, compared with the control.

Under conditions inducing changes in ES cells (MEF+/LIF− and MEF−/LIF−), lignin was added to ES cells and then changes in the expressions of differentiation markers and undifferentiation markers in ES cells were analyzed. Results showed that when lignin was added to ES cells under the condition of MEF+/LIF−, a notable change was observed in colony morphology (Fig. 2a). In gene expression analysis by real-time PCR, the expressions of the undifferentiation markers, Nanog and Rex1 were notably suppressed. In contrast, the expressions of the neuroectodermal markers, Otx2 and Sox1 were significantly promoted in a concentration-dependent manner (Fig. 2b, Fig. S1). The expressions of endodermal markers (Foxa2 and Sox17) and mesodermal markers (Flk1 and Brachyury) were hardly observed. Also, no effect of lignin on ES cells was detected (data not shown). Therefore, the data of endodermal and mesodermal markers were omitted in Fig. 2b and Fig. S1.

**Figure 3:**
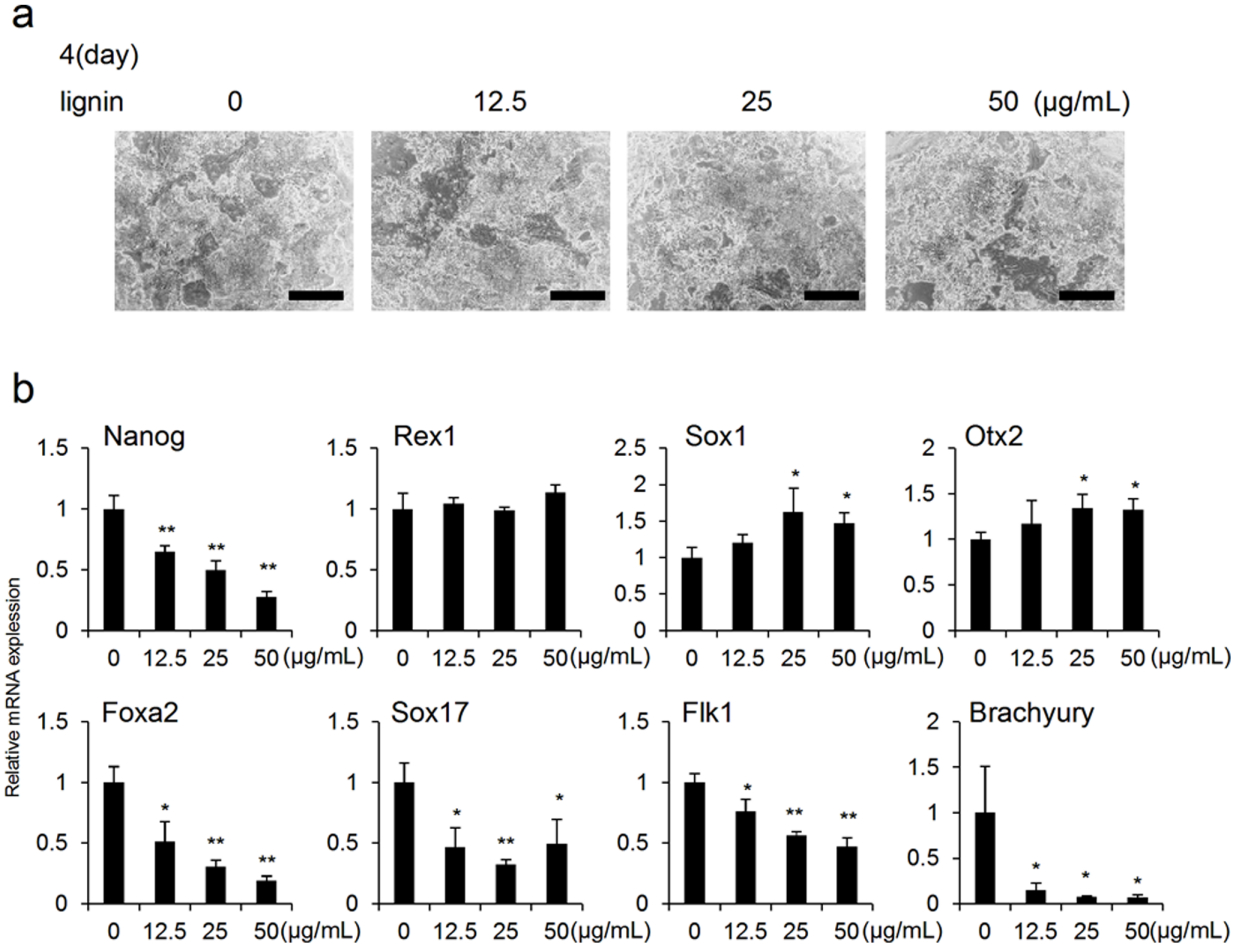
Analysis of the effects of lignin on ES cells cultured in absence of MEF and LIF (MEF−/LIF−). (a) Microscopic images of ES cells on day 4 of culture. ES cells were cultured with lignin (12.5, 25 and 50 µg/mL). (b) Gene expression analysis by real-time PCR. Changes in the expressions of differentiation marker genes associated with the addition of lignin were analyzed on day 4 of culture. The expression level of each marker gene was normalized to control (0 µg/mL). Scale bar: a = 200 µm. Data are expressed as the mean ± SD of the three experiments. *P<0.05, **P<0.01, compared with the control.

Furthermore, we conducted the same experiment as the above described method under the condition of MEF−/LIF−. Results showed that the expression of Nanog was suppressed on day 4 and the expressions of the neuroectodermal markers, Sox1 and Otx2 were slightly increased (Fig. 3b), whereas the expressions of endodermal markers (Foxa2 and Sox17) and mesodermal markers (Flk1 and Brachyury) were significantly suppressed on days 4 and 6 (Fig. 3b, Fig. S2).

Based on the above results, lignin was shown to inhibit the undifferentiated state of mouse ES cells and promote differentiation into neuroectodermal cells, whereas it was shown to suppress differentiation into endodermal cells and mesodermal cells.

### 2. Lignin Inhibits the Wnt Signaling Pathway

Previous studies have shown that Wnt signaling plays an important role in maintaining the undifferentiation state of ES cells [11]. In addition, it has been reported that inhibiting Wnt signaling promotes the differentiation of ES cells into neuroectodermal cells and, in contrast, suppresses differentiation into endodermal and mesodermal cells [12].

**Figure 4:**
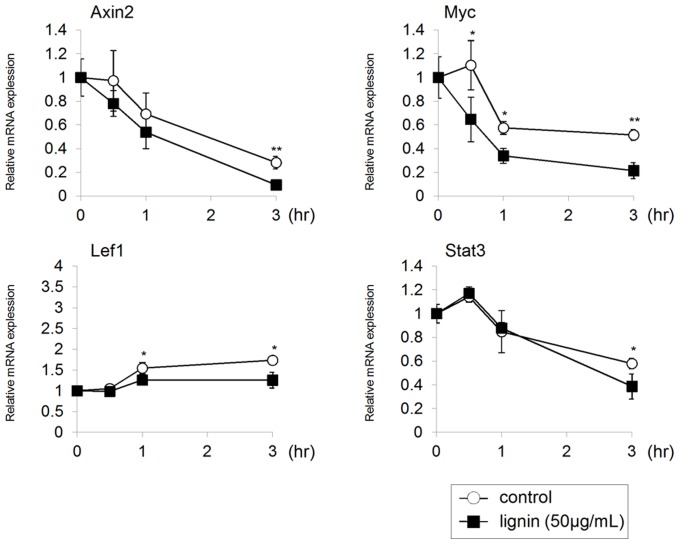
Analysis of the effects of lignin on Wnt/β catenin signaling of ES cells. Gene expression analysis by real-time PCR. ES cells cultured in an undifferentiated state were separated from MEF and we added lignin (50 µg/mL) to ES cells. After lignin was added to ES cells, total RNA were collected after 0, 0.5, 1 and 3 hours, and gene expression analysis was conducted by real-time PCR. The expression level of each marker gene was normalized to time 0. Data are expressed as the mean ± SD of the three pone.0066376.g005.tifexperiments. *P<0.05, **P<0.01, compared with the control.

The above experiments demonstrated that lignin causes effects on ES cells similar to those yielded when Wnt signaling is inhibited. Therefore, we examined the effects of lignin on the expression of the Wnt target genes of ES cells. In this study, we added lignin to ES cells and collected total RNA after 0, 0.5, 1 and 3 hours to analyze the expressions of Wnt signaling target genes by real-time PCR. Results showed that the expressions of the direct target genes of Wnt signaling, Axin2, Myc, and Lef1, were suppressed in the Lignin-added group compared to the control group. The expression of Stat3, which acts downstream of Wnt signaling to maintain an undifferentiated state of ES cells [11], was also suppressed (Fig. 4). Considering these results, it has been suggested that lignin may inhibit Wnt signaling in ES cells.

**Figure 5:**
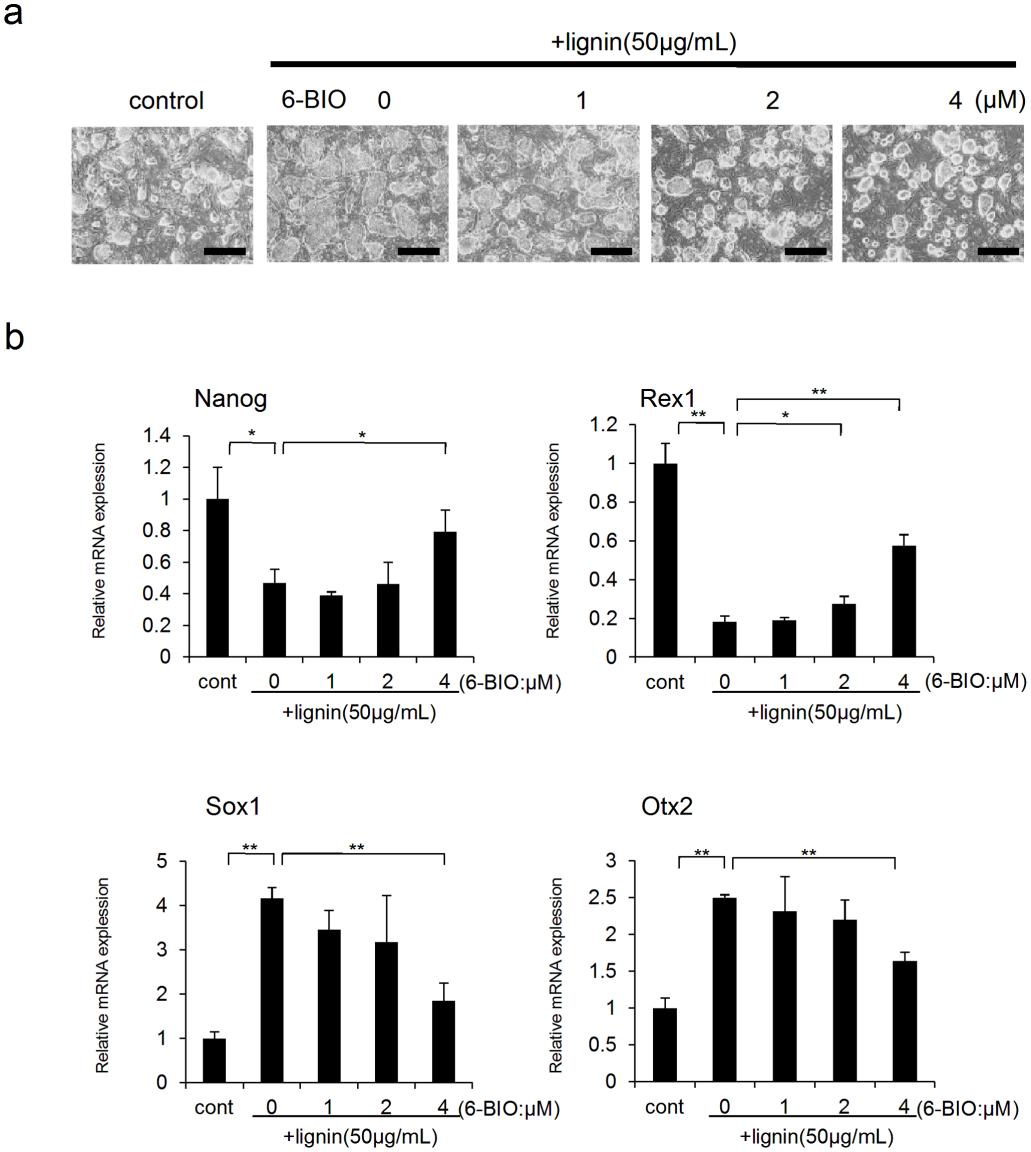
Effects of lignin and 6-BIO on differentiation of ES cells. (a) Microscopic images of ES cells on day 4 of induction. ES cells were cultured with lignin (50 µg/mL) and various concentrations of 6-BIO (1, 2 and 4 µM). (b) Gene expression analysis by real-time PCR. Changes in the expressions of differentiation marker genes associated with the addition of lignin and 6-BIO were analyzed on day 4 of culture. The expression level of each marker gene was normalized to control. Scale bar: a = 200 µm Data are expressed as the mean ± SD of the three experiments. *P<0.05, **P<0.01, compared with the control.

In order to more specifically investigate the above results, we examined if lignin-induced suppression of Wnt signaling can be restored using a GSK-3β inhibitor (6-BIO) that specifically activates Wnt signaling. It was shown that undifferentiated ES cell-like colonies were confirmed when 6-BIO was added to ES cells whose colony morphology was changed because the undifferentiated state was suppressed by lignin (Fig. 5a). In real-time PCR analysis, it was also revealed that lignin-induced effects, which promote ES cells to differentiate into neuroectodermal cells, were inhibited by 6-BIO in a concentration-dependent manner (Fig. 5b). From the above results, we concluded that lignin-induced suppression of the undifferentiated state and promotion of differentiation into neuroectodermal cells could be attributed to the inhibition of Wnt signaling in ES cells.

### 3. Establishment of a Method for Differentiation Induction of ES Cells into Ocular Cells by Lignin

The development of eyes originates from optic vesicles: projections yielded on both sides of the interbrain, which is a part of the brain derived from the neural tube. Optic vesicles induce a lens by acting on the epidermis, producing an optic cup that differentiates into such ocular tissues as the neural retina and retinal pigment epithelium (RPE). It has been shown that Wnt signaling must be inhibited for the development of eyes from the neural tube [13]. It is also known that pluripotent stem cells can be effectively promoted to differentiate into retinal tissues when Wnt signaling is inhibited [14]. Based on these findings, we examined if it is possible to establish a differentiation inducement system that effectively promotes differentiation of ES cells into ocular cells using lignin.

Yamane et al. previously established a differentiation inducement system to produce melanocytes through a stage of neural crest cells (neural tube-derived) using mouse ES cells [15], [16]. Using their differentiation inducement system, we analyzed the effects of lignin on differentiation.

**Figure 6:**
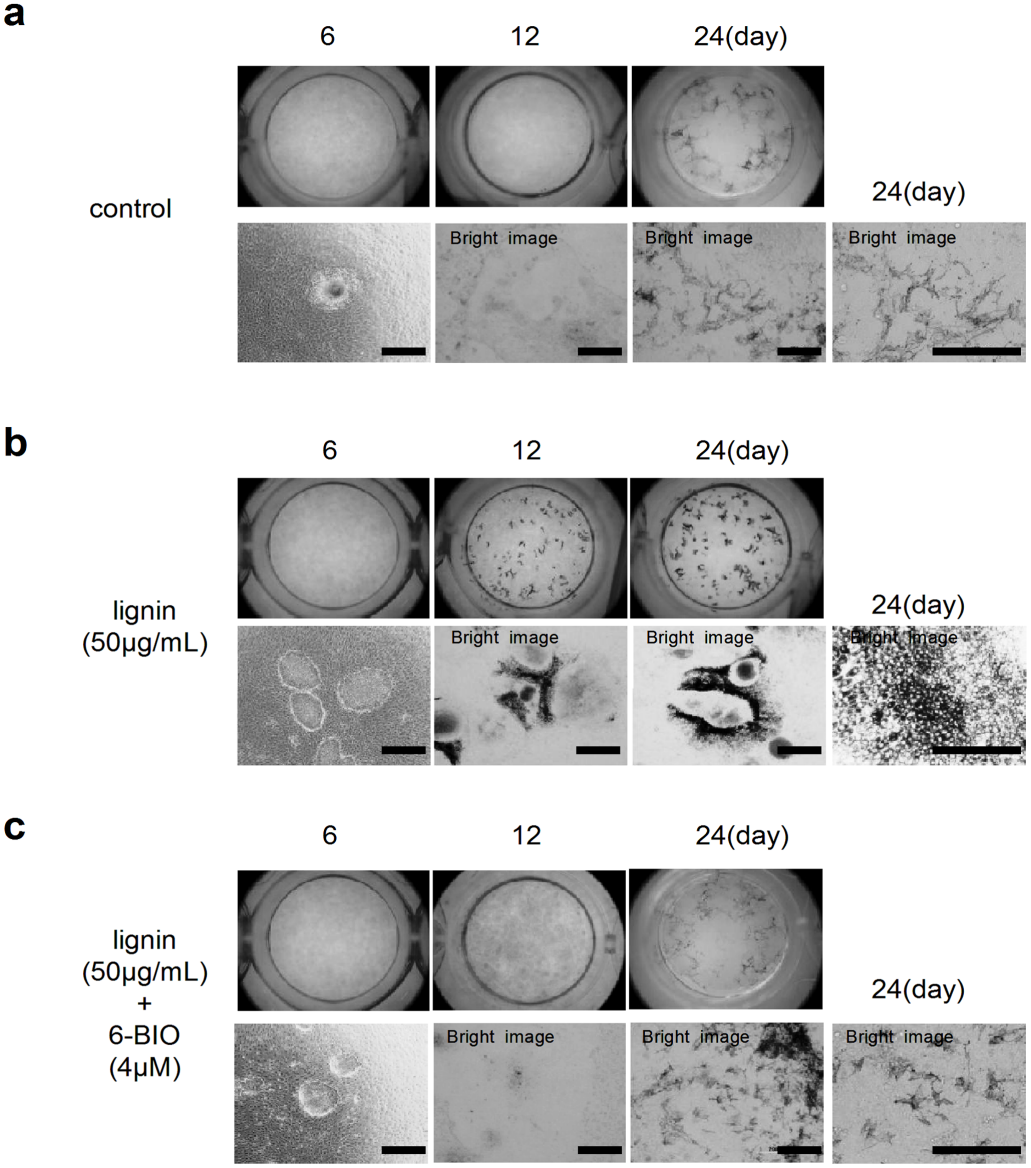
Effects of lignin and 6-BIO on the differentiation of ES cells into ocular cells. (a) Microscopic images of ES cells (Control). ES cells were seeded on ST2 cells and cultured in a melanocyte differentiation inducement medium. (b) Microscopic images of ES cells (lignin-added group). ES cells were cultured under lignin-added condition (50 µg/mL) in an ST2 cell-seeded melanocyte differentiation inducement medium. (c)Microscopic images of ES cells (lignin- and 6-BIO-added group). ES cells were cultured with lignin (50 µg/mL) and 6-BIO (4 µM) in a ST2 cell-seeded melanoctye differentiation inducement medium. Scale bar: a,b,c = 200 µm.

First, we induced differentiation using ES cells based on a study by Yamane et al. Microscopic observations showed that ES cells formed colonies on ST2 cells (feeder cells) by day 6 of induction and pigmented melanocytes appeared near the colonies around day 18, followed by continuous proliferation until day 24 (Fig. 6a) [16]. Melanocytes showed the dendritic morphology typical of skin melanocytes.

Next, we added 50 µg/mL of lignin to the differentiation inducement system (Fig. 6b). The colony morphology of the Lignin-added group was markedly different from that of the control group on day 6. Furthermore, pigmented structures appeared around the colonies on day 12. These pigmented cells showed polygonal cell structures containing relatively large melanin granules (Fig. 6b and Fig. S3a), suggesting that these cells constitute RPE. Furthermore, these cells were positive for RPE65 (an RPE cell marker), Keratin (an epidermal lineage specific marker), ZO-1 (tight junction marker) (Figs. S3b-d). We also confirmed an alpha-B crystallin-positive structure, which is a lens marker, adjacent to RPE (Fig. S3e).

Unlike the control group, skin melanocytes did not appear on day 24 of induction in the Lignin-added group.

**Figure 7:**
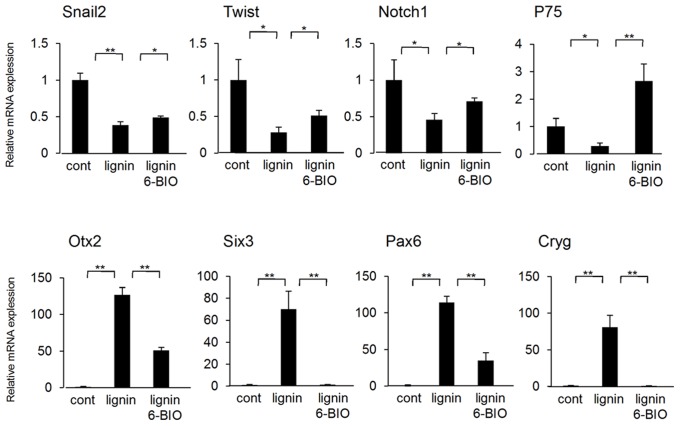
Effects of lignin and 6-BIO on the differentiation of ES cells into ocular cells (gene expression analysis). Gene expression analysis by real-time PCR. On day 6 of induction, changes in the expressions of neural crest cell marker genes and eye development marker genes were analyzed. The expression level of each marker gene was normalized to control. Data are expressed as the mean ± SD of the three experiments. *P<0.05, **P<0.01, compared with the control.

When 6-BIO was added to the Lignin-added group, RPE-like cells did not appear on day 12, but skin melanocytes appeared on day 24 of induction (Fig. 6c). We collected ES cells cultured under each condition on day 6 of induction and analyzed the expression of differentiation markers by real-time PCR (Fig. 7). Results showed that the expression of the neural crest cell markers, Snail2, Twist, Notch1, and P75 were significantly suppressed by lignin and were then recovered by the addition of 6-BIO. In contrast, the expressions of the eye development markers, Otx2, Six3, Pax6, and Cryg (lens marker) were significantly promoted by lignin and were then recovered by the addition of 6-BIO (Fig. 7).

These results suggest that the Wnt signaling pathway considered to be necessary for the development of neural crest cells and melanocytes were inhibited by adding lignin in the early stages of induction. Consequently, ES cells were induced to differentiate into eye-like structures having a RPE and lens. Actually, we confirmed that Wnt ligands, Wnt1, 3a, and 8a involved in the development of neural crest cells and melanocytes were transiently increased in the early stages of induction in the melanocyte differentiation inducement system (control group) (Fig. S4).

Based on the above results, when IWR-1, a Wnt/β catenin signaling pathway-specific inhibitor, was added instead of lignin to the melanocyte differentiation inducement system, colony morphology was changed on day 6 as with lignin, and eye-like structures having a RPE were formed on day 12 (Fig. S5b).

Taken together, we discovered that lignin has a powerful effect that suppresses Wnt signaling in ES cells and that it is possible to effectively promote ES cells to differentiate into neuroectodermal cells by lignin. Furthermore, we established a method to effectively promote ES cells to differentiate into ocular cells.

### 4. Application of Lignin to Other Differentiation Inducement Systems

Previous studies have shown that the suppression of Wnt signaling is effective in a neural cell differentiation inducement system for ES cells [17]. Furthermore, technology to induce mature neural cells has been established, in which ES cells are co-cultured with various stromal cells in serum-free culture [18], [19]. We examined if lignin could promote differentiation efficiency in the neural differentiation inducement system, using the presented suppressing effects of lignin on Wnt signaling. More specifically, we established a neural cell differentiation inducement system, based on the system established by previous studies, and examined the effect of lignin on differentiation inducement into neural cells using this system. As a result, nervous-specific dendrites were found in colonies of the Lignin-added group on day 8 of induction (Fig. S6a). When immunostaining was performed on dendrite-like structures in the Lignin-added group, Neurofilament and MAP2, a neural differentiation marker, was found to be positive (Figs. S6b and c). Although neural dendrites were also observed in the control group on day 12 of induction, the number of dendrites tended to be larger in the Lignin-added group (Fig. S6a). On day 8, we collected total RNA and analyzed the expression of the neural cell differentiation marker genes, Pax6, Tuj-1, and MAP2. Results showed that the expression of each marker gene was significantly promoted by the additions of lignin (Fig. S6d). Furthermore, we confirmed that the expression of Myc is downregulated by adding lignin using the differentiation inducement system (Fig. S7). Therefore, it was confirmed that lignin is also effective in promoting differentiation in the neural cell differentiation inducement system for ES cells.

## Discussion

For the first time, we succeeded in elucidating the effects of lignin on ES cells. First, we evaluated the effect of lignin on the maintenance of an undifferentiated state and differentiation induction in ES cells. ES cells maintained an undifferentiated state when cultured under the condition of MEF+/LIF+, whereas differentiation proceeded under the condition of MEF+/LIF− and MEF−/LIF− (Fig. 1). Previous studies have shown that MEF secretes factors crucial for maintaining the undifferentiated state of ES cells [20]. It has also been reported that LIF is an important factor for maintaining the undifferentiated state of ES cells [21]. In this study, it was confirmed that when lignin was added to ES cells cultured under the condition of MEF+/LIF− and MEF−/LIF−, the expression of undifferentiation markers was downregulated, and the expression of neuroectodermal markers was promoted (Figs. 2, 3, Figs. S1, 2). As a reason that the effect of lignin was abated under the condition of MEF−/LIF−, it was considered that the expressions of undifferentiation markers, Rex1 and Nanog, were spontaneously, notably downregulated without lignin, and the expressions of neuroectodermal markers, Sox1 and Otx2, were spontaneously, notably upregulated with lignin under the condition of MEF−/LIF− (Fig. 1b). Since lignin was added under the condition, it was likely that no further change of the expressions was observed about undifferentiation and neuroectodermal markers (Fig. 3, Fig. S2). We believe that Nanog was downregualted and Rex1 was not downregulated on day 4 because Rex1 had been decreased to the full extent as of day 4, and therefore the expression of Rex1 was not decreased any further even after lignin was added.

Lignin also downregulated the expression of Wnt target genes (Fig. 4). In this experiment, time 0 was defined as standard value for comparing the expression level of each gene. In order to analyze direct effects of lignin on ES cells, undifferentiated ES cells cultured on MEF were treated with trypsin to separate from MEF, and then lignin was added. We believe that the changes in gene expressions between time 0 and time 3 hour in the control group were caused when ES cells became single cells after being separated from MEF. The Wnt signaling pathway plays an important role in various stages from early development to morphogenesis. For example, the Wnt/β catenin pathway (canonical Wnt signaling), PCP (planar cell polarity signaling), and Wnt/Ca^2+^ pathway are well-known Wnt signaling pathways. In this study, the effect of lignin was counteracted by 6-BIO, which specifically activates the Wnt/β catenin signaling pathway (Figs. 5, 6); therefore, lignin was considered to suppress the Wnt/β catenin signaling pathway.

Previous studies have also shown that the suppression of Wnt signaling is indispensable for the development of eyes [13]. Thus, we explored a method to induce the differentiation of ES cells into eye-like structures using the Wnt signaling-suppressing effect of lignin. We succeeded in establishing a method that effectively induces the differentiation of ES cells into ocular cells by adding lignin to a melanocyte differentiation inducement system. The structures induced by our inducement system were comparable to the eye-like structures formed in the eye-like structure inducement system for ES cells, established by Hirano et al. [22]. According the study by Hirano et al., when ES cells were seeded on PA6 (feeder cells) and cultured using differentiation inducing medium, expression of a marker of eye development, Pax6, was increased and highly pigmented cells having an RPE-characteristic hexagonal shape appeared on day 11 as seen in our study. Hirano et al. also reported that the eye-like structures were positive for alpha-B crystallin (a lens marker). There was no major difference in the differentiation inducing medium between the method by Hirano et al. and ours. A major difference is that Hirano et al. used PA6 as feeder cells, not ST2. Interestingly, a recent study has shown that PA6 secrets Wnt5a which is an inhibitor of canonical Wnt signaling [23].

Furthermore, IWR-1, a Wnt/β catenin signaling pathway-specific inhibitor, exhibited the same eye-like structure inducing effect as lignin (Fig. S5). These findings led us to conclude that the eye-like structure inducing effect of lignin could be attributed to the suppressing effect of the Wnt/β catenin signaling pathway.

In the experiment using the differentiation inducement system of ES cells into neural cells, lignin also promoted the differentiation (Fig. S6). The expression of Myc (a Wnt target gene) was significantly downregulated (Fig. S7). Previous study, in fact, demonstrated that central nervous system progenitors are excessively produced when Myc is absent in the early development of Xenopus [24]. Taken these findings together, it was suggested that lignin induces inhibition of Wnt signaling and thereby promotes the differentiation of ES cells into neural cells.

Meanwhile, the mechanism underlying the suppressing effect of lignin on the Wnt/β catenin signaling pathway is still unknown. Lignin has a large, complicated structure in which coniferyl alcohol and lignan (progenitors of lignin) are polymerized. We believe that the effects of lignin on ES cells may be attributed to its complicated structure. In fact, although we examined other compounds such as various kinds of lignan (including arctiin and matairesinol) and coniferyl alcohol by the same method, no effect similar to lignin was detected (data not shown). Wnt signaling is transmitted to the downstream as Wnt ligand is bound to receptors on the cell membrane. Considering that lignin is unlikely to penetrate a plasma membrane due to its high molecular property, it is conceivable that lignin inhibited binding of the receptors and Wnt ligand on cell membrane. This is also supported by the finding that lignin became ineffective by addition of 6-BIO which is a cell-permeable, low-molecular compound and also a GSK-3β inhibitor (Wnt signal activator). Further study is required to elucidate a more specific mechanism underlying the suppressing effect of lignin on the Wnt signaling pathway of ES cells.

Our study demonstrated that lignin exhibits a remarkable suppressing effect on Wnt/β catenin in ES cells, and that ES cells can be effectively induced to ocular cells and neural cells by the inhibitory effect of lignin. Once the retina andRPE of mammals suffer damage, they do not spontaneously regenerate themselves. Since there is no appropriate treatment for retinal degeneration including retinal pigmentary degeneration, many patients end up losing their sight. Such patients with impaired vision have hope for the development of a new treatment by regenerative medicine. In addition, every year, there are a growing number of patients suffering spinal injuries. Severe spinal injury causes crippled extremities, leading patients to be bedridden or confined to a wheelchair for their entire life. At present, no effective medical treatment is available for recovering the function lost by spinal injury since it is extremely difficult to regenerate nerves in the central nervous system. Thus, regenerative treatment for spinal injury is also strongly expected to become clinically available. Lignin used in this study is a natural material commonly found in plants and can be produced at a relatively low cost in large volumes. If it becomes possible to easily and quickly produce cells such as retinal cells, RPE, and neural cells using lignin, regenerative medical treatment using these cells will become widely available in clinical settings.

## Supporting Information

Figure S1 **Analysis of the effects of lignin on ES cells cultured in presence of MEF and absence of LIF (MEF+/LIF−).** Gene expression analysis by real-time PCR. Changes in the expressions of differentiation marker genes associated with the addition of lignin were analyzed on day 6 of culture. The expression level of each marker gene was normalized to control (0 µg/mL). Data are expressed as the mean ± SD of the three experiments. *P<0.05, **P<0.01, compared with the control.
(TIF)

Figure S2 **Analysis of the effects of lignin on ES cells cultured in absence of MEF and LIF (MEF−/LIF−).** Gene expression analysis by real-time PCR. Changes in the expression of differentiation marker genes associated with the addition of lignin were analyzed on day 6 of culture. The expression level of each marker gene was normalized to control (0 µg/mL). Data are expressed as the mean ± SD of the three experiments. *P<0.05, **P<0.01, compared with the control.
(TIF)

Figure S3 **Expression of eye-specific markers in the induced eye-like structures induced from lignin-added ES cells.** (a) Higher-magnification image of the RPE like structure induced from ESCs after 12-day culture. (b)–(e) Immunostaining of eye- like structures. Eye- like structures induced from ESCs after 12-day culture were stained with antibodies against RPE65 (b; red), Keratin (c; red) and ZO-1 (d; green) and alpha-B crystallin (e; green), and nuclei were stained with DAPI solution (blue). Scale bar: a = 50 µm, b,d = 200 µm, c,e = 100 µm.
(TIF)

Figure S4 **Changes in the expressions of various Wnt ligands associated with melanocyte differentiation.** Gene expression analysis by real-time PCR. total RNA were collected on days 0, 3, 6, 9, 12, 15, 18, 21 and 24 to analyze the changes in the expressions of each Wnt ligand. The expression level of each marker gene were normalized to that on day 0. Data are expressed as the mean ± SD of the three experiments.
(TIF)

Figure S5 **Effects of IWR-1 on the differentiation of ES cells into ocular cells.** (a) Microscopic images of ES cells (control). ES cells were seeded on ST2 cells and cultured in a melanocyte differentiation inducement medium. (b) Microscopic images of ES cells (IWR-1-added group). ES cells were seeded on ST2 cells and cultured in a melanocyte differentiation inducement medium under IWR-1 (10 µM)-added condition. Scale bar: a,b = 200 µm.
(TIF)

Figure S6 **Effects of lignin on the differentiation of ES cells into neural cells.** (a) Microscopic images of ES cells (control and lignin-added groups). ES cells were seeded on ST2 cells and cultured in a neural cell differentiation induction medium. (b,c) Immunostaining of neural cells induced from lignin-added ES cells. Neural cells obtained from ESCs after 8-day culture were stained with antibodies against Neurofirament (b; red) and MAP2 (c; green), and nuclei were stained with DAPI solution (blue). (d) Gene expression analysis by real-time PCR. Changes in the expression of neural cell differentiation markers associated with the addition of lignin were analyzed on day 8 of induction. The expression level of each marker gene was normalized to control. Scale bar: a,b,c = 200 µm. Data are expressed as the mean ± SD of the three experiments. *P<0.05, **P<0.01, compared with the control.
(TIF)

Figure S7 **Effects of lignin on Wnt/β catenin signaling in the neural differentiation system of ES cells.** Gene expression analysis by real-time PCR. Change in the expression of Myc associated with the addition of lignin was analyzed on days 4 and 8 of induction. The expression level of Myc was normalized to control on day 4. Data are expressed as the mean ± SD of the three experiments. **P<0.01, compared with the control.
(TIF)
