# Supplementary figures and images for: Lignin Induces ES Cells to Differentiate into Neuroectodermal Cells through Mediation of the Wnt Signaling Pathway
Source: PLoS One. 2013 Jun 21;8(6):e66376. doi: 10.1371/journal.pone.0066376 (PMC3689838; doi:10.1371/journal.pone.0066376)

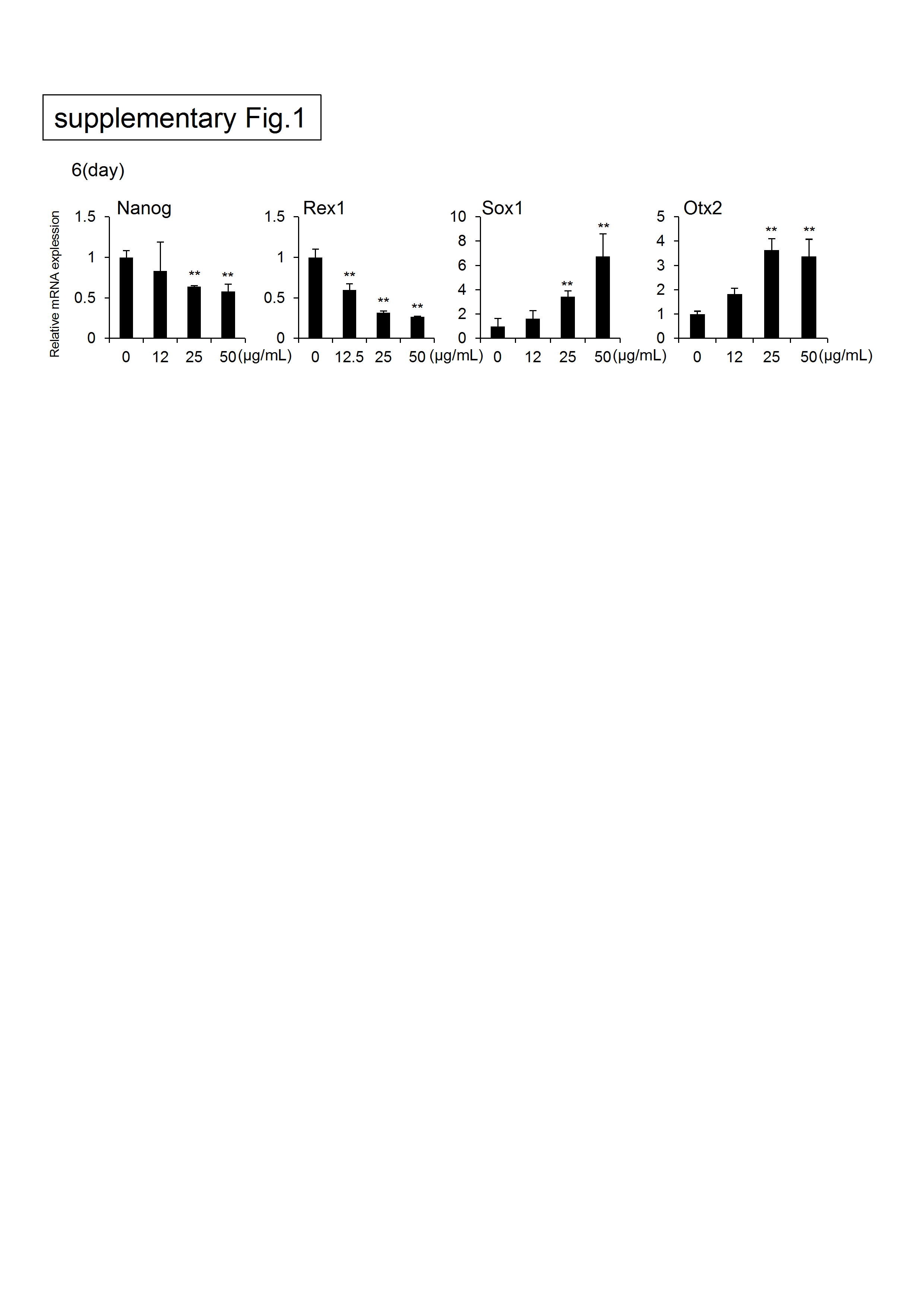

Supplement: Figure S1 — Analysis of the effects of lignin on ES cells cultured in presence of MEF and absence of LIF (MEF+/LIF−). Gene expression analysis by real-time PCR. Changes in the expressions of differentiation marker genes associated with the addition of lignin were analyzed on day 6 of culture. The expression level of each marker gene was normalized to control (0 µg/mL). Data are expressed as the mean ± SD of the three experiments. *P<0.05, **P<0.01, compared with the control. (TIF) [file pone.0066376.s001.tif]

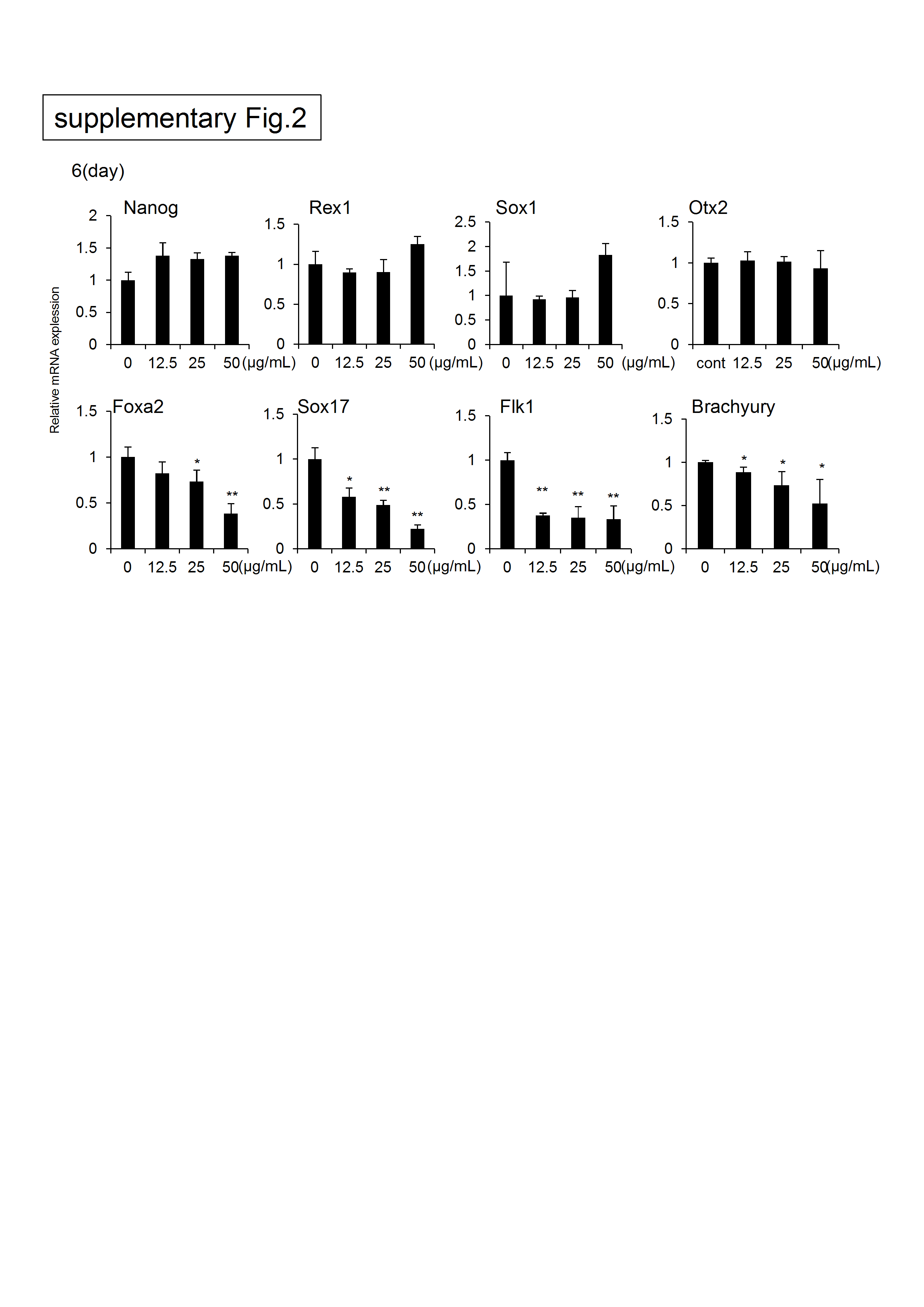

Supplement: Figure S2 — Analysis of the effects of lignin on ES cells cultured in absence of MEF and LIF (MEF−/LIF−). Gene expression analysis by real-time PCR. Changes in the expression of differentiation marker genes associated with the addition of lignin were analyzed on day 6 of culture. The expression level of each marker gene was normalized to control (0 µg/mL). Data are expressed as the mean ± SD of the three experiments. *P<0.05, **P<0.01, compared with the control. (TIF) [file pone.0066376.s002.tif]

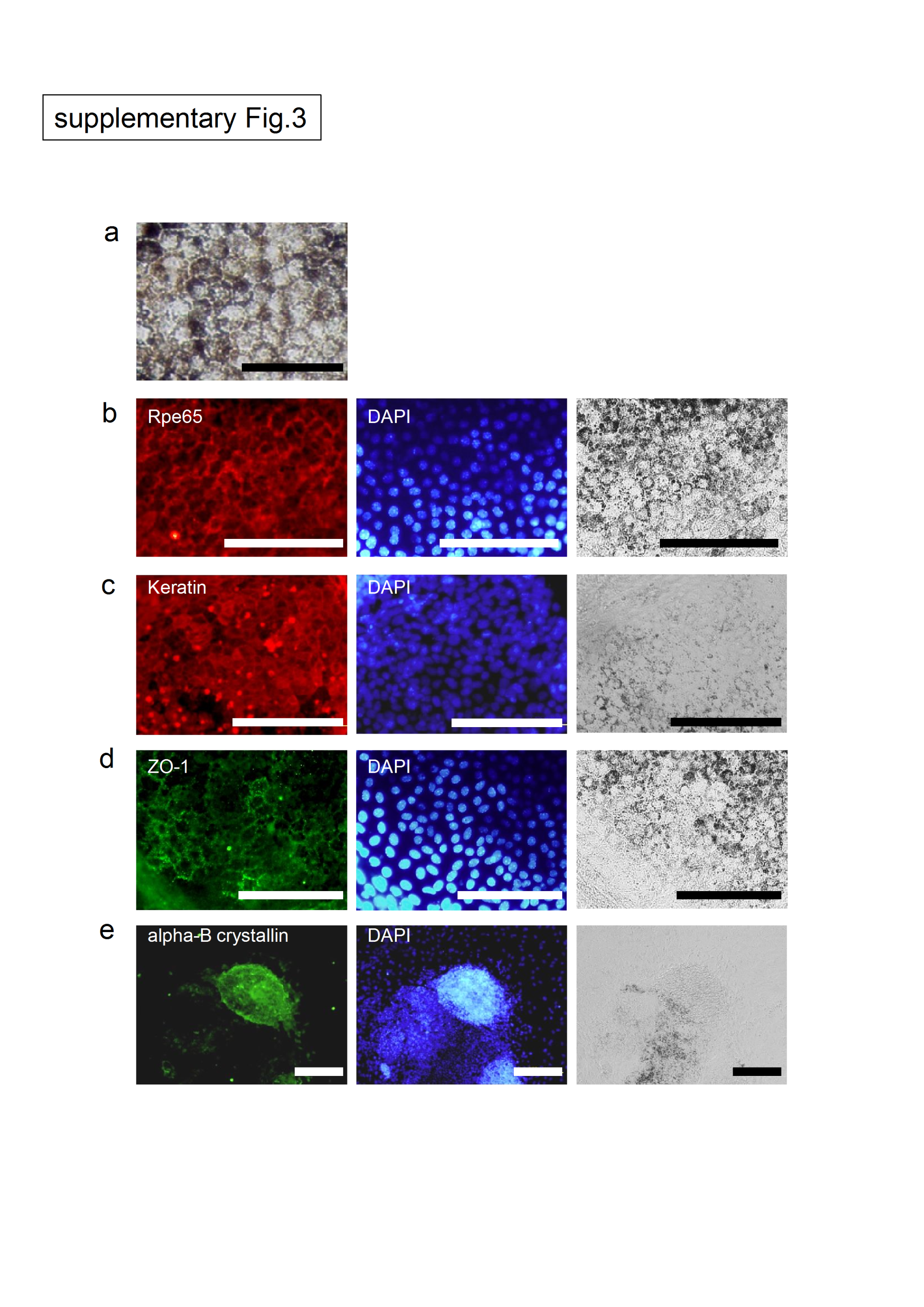

Supplement: Figure S3 — Expression of eye-specific markers in the induced eye-like structures induced from lignin-added ES cells. (a) Higher-magnification image of the RPE like structure induced from ESCs after 12-day culture. (b)–(e) Immunostaining of eye- like structures. Eye- like structures induced from ESCs after 12-day culture were stained with antibodies against RPE65 (b; red), Keratin (c; red) and ZO-1 (d; green) and alpha-B crystallin (e; green), and nuclei were stained with DAPI solution (blue). Scale bar: a = 50 µm, b,d = 200 µm, c,e = 100 µm. (TIF) [file pone.0066376.s003.tif]

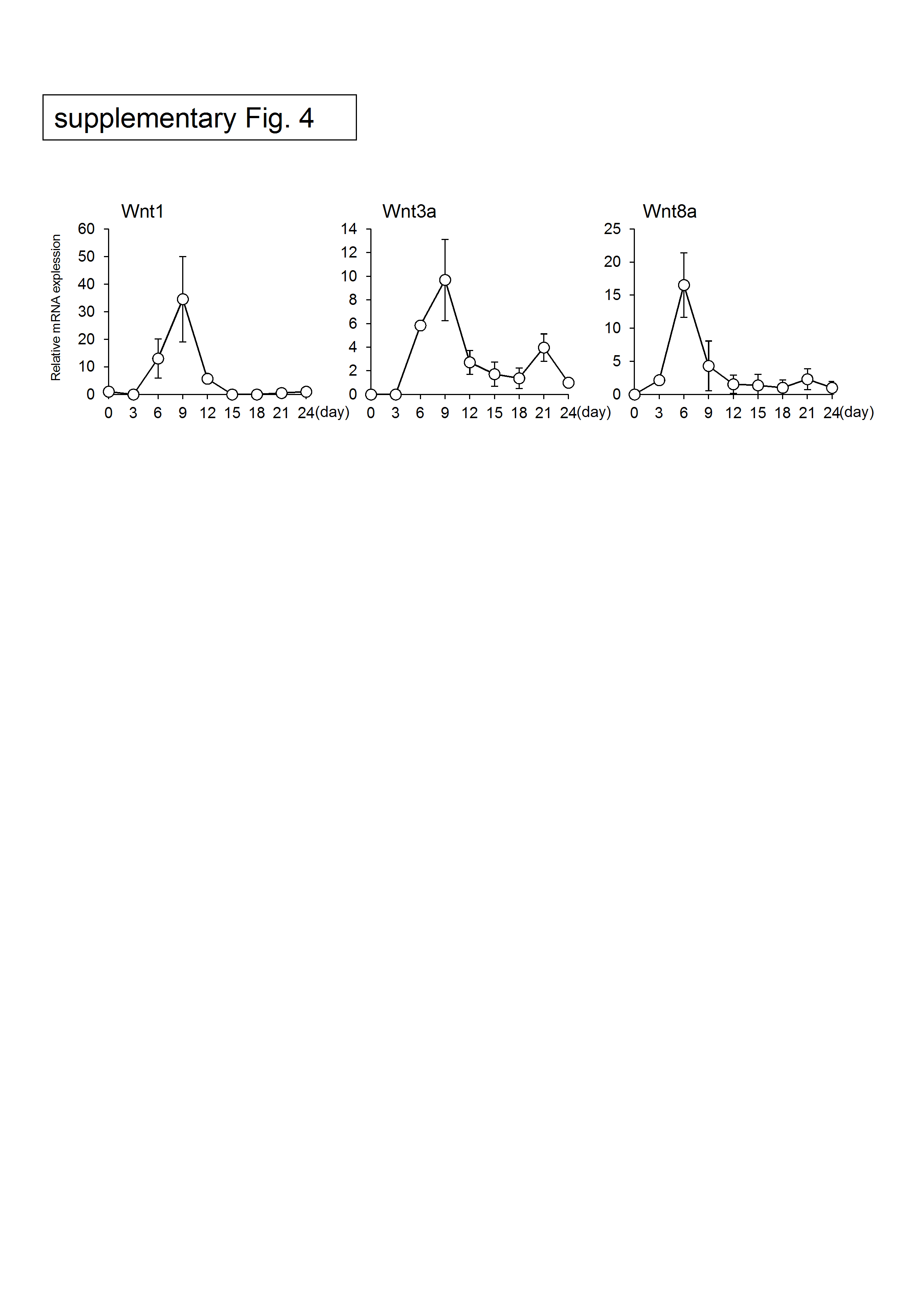

Supplement: Figure S4 — Changes in the expressions of various Wnt ligands associated with melanocyte differentiation. Gene expression analysis by real-time PCR. total RNA were collected on days 0, 3, 6, 9, 12, 15, 18, 21 and 24 to analyze the changes in the expressions of each Wnt ligand. The expression level of each marker gene were normalized to that on day 0. Data are expressed as the mean ± SD of the three experiments. (TIF) [file pone.0066376.s004.tif]

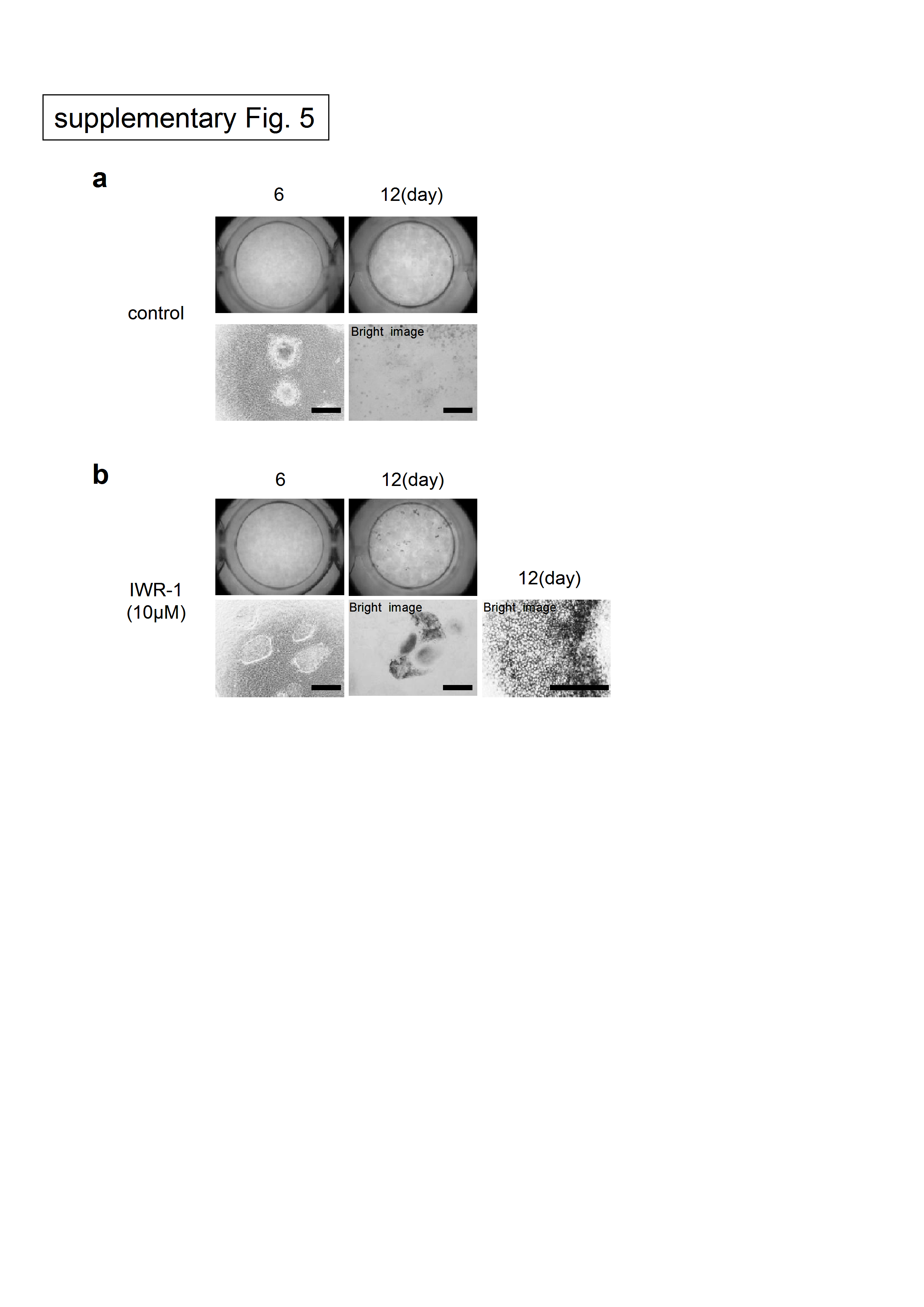

Supplement: Figure S5 — Effects of IWR-1 on the differentiation of ES cells into ocular cells. (a) Microscopic images of ES cells (control). ES cells were seeded on ST2 cells and cultured in a melanocyte differentiation inducement medium. (b) Microscopic images of ES cells (IWR-1-added group). ES cells were seeded on ST2 cells and cultured in a melanocyte differentiation inducement medium under IWR-1 (10 µM)-added condition. Scale bar: a,b = 200 µm. (TIF) [file pone.0066376.s005.tif]

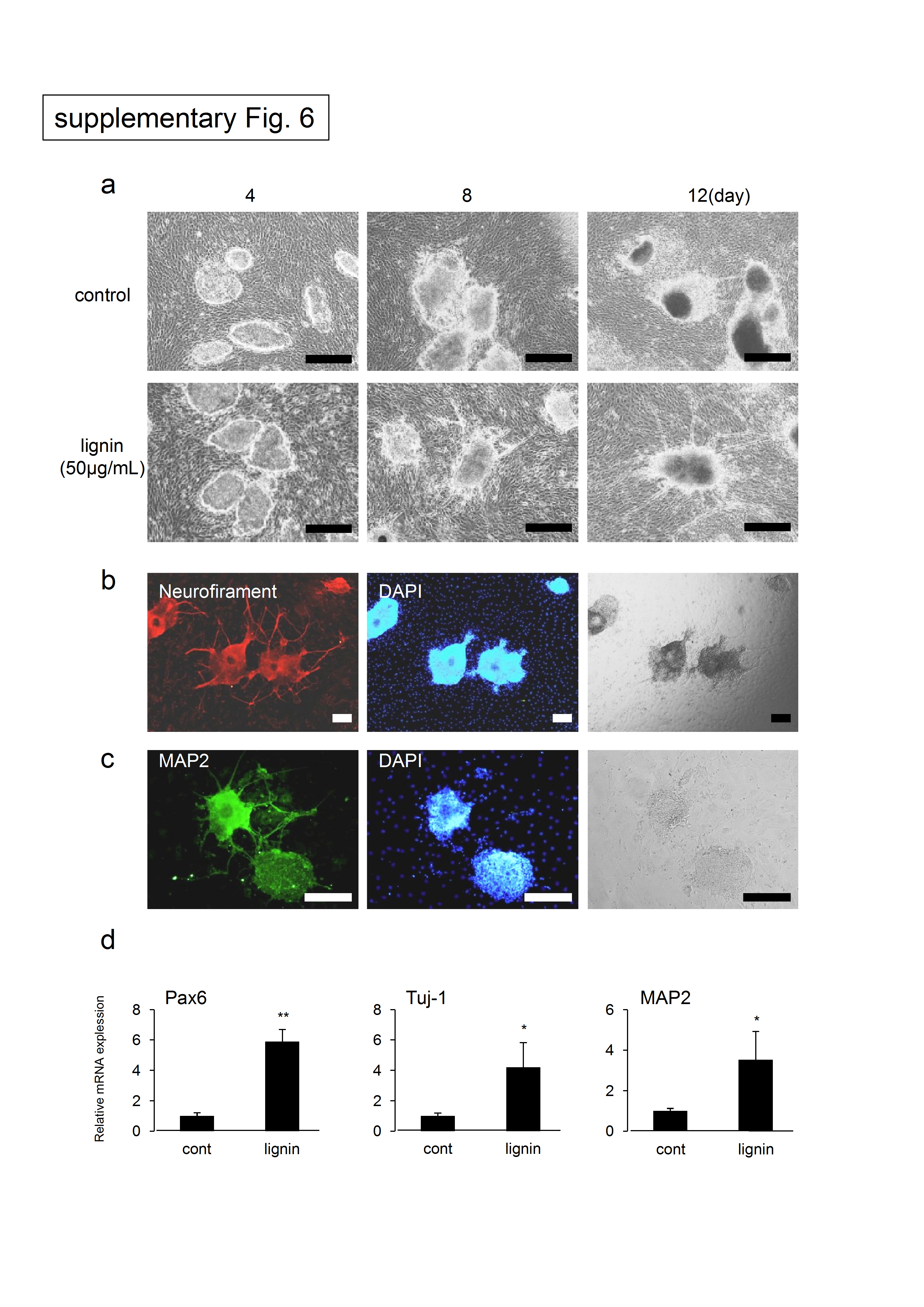

Supplement: Figure S6 — Effects of lignin on the differentiation of ES cells into neural cells. (a) Microscopic images of ES cells (control and lignin-added groups). ES cells were seeded on ST2 cells and cultured in a neural cell differentiation induction medium. (b,c) Immunostaining of neural cells induced from lignin-added ES cells. Neural cells obtained from ESCs after 8-day culture were stained with antibodies against Neurofirament (b; red) and MAP2 (c; green), and nuclei were stained with DAPI solution (blue). (d) Gene expression analysis by real-time PCR. Changes in the expression of neural cell differentiation markers associated with the addition of lignin were analyzed on day 8 of induction. The expression level of each marker gene was normalized to control. Scale bar: a,b,c = 200 µm. Data are expressed as the mean ± SD of the three experiments. *P<0.05, **P<0.01, compared with the control. (TIF) [file pone.0066376.s006.tif]

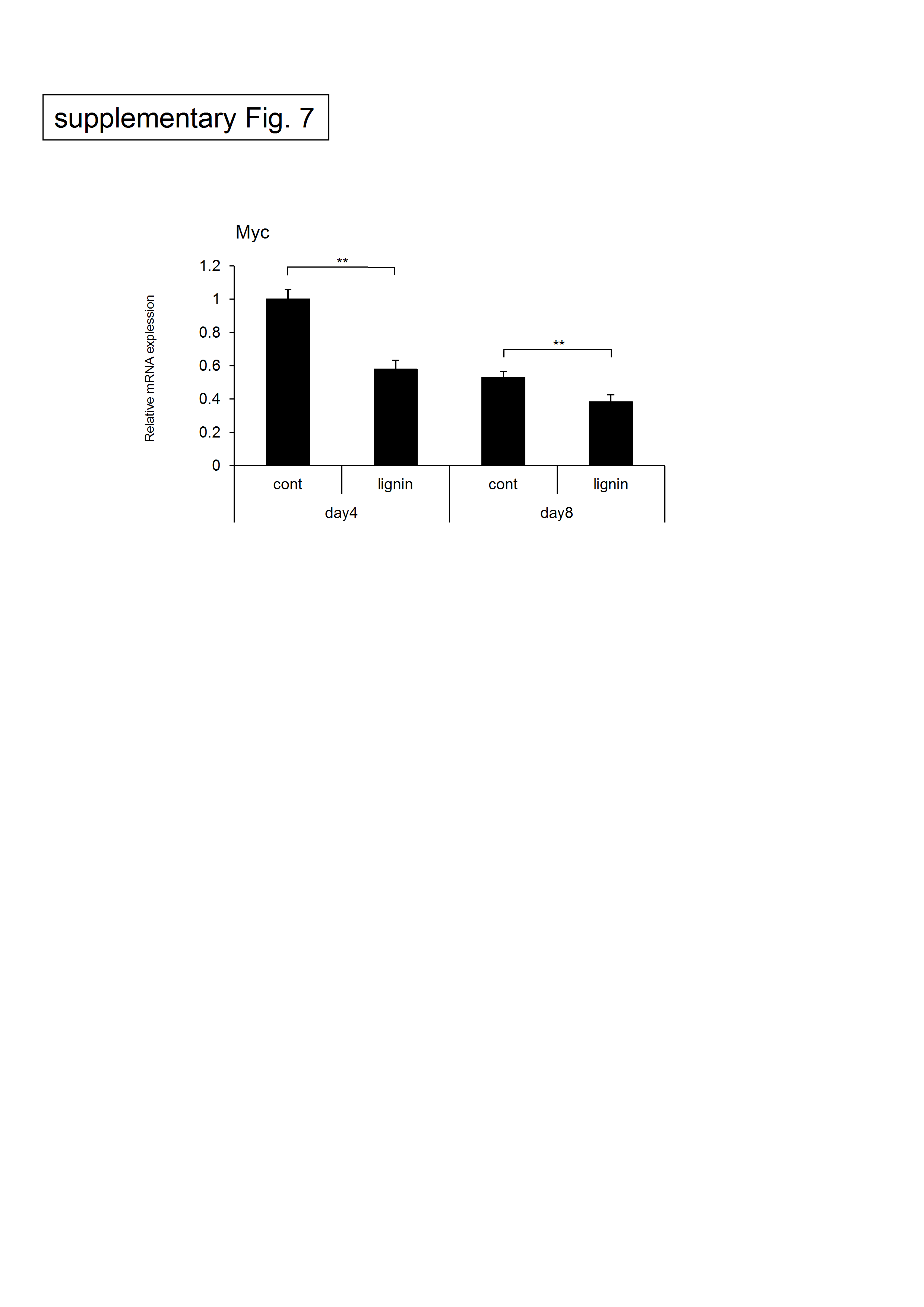

Supplement: Figure S7 — Effects of lignin on Wnt/β catenin signaling in the neural differentiation system of ES cells. Gene expression analysis by real-time PCR. Change in the expression of Myc associated with the addition of lignin was analyzed on days 4 and 8 of induction. The expression level of Myc was normalized to control on day 4. Data are expressed as the mean ± SD of the three experiments. **P<0.01, compared with the control. (TIF) [file pone.0066376.s007.tif]
